# RT-qPCR study of COX-1 and -2 genes in oral surgical model comparing single-dose preemptive ibuprofen and etoricoxib: A randomized clinical trialy

**DOI:** 10.4317/jced.56447

**Published:** 2020-04-01

**Authors:** Assis-Filipe Medeiros-Albuquerque, Cristiane-Sá Roriz-Fonteles, José-Jackson do Nascimento-Costa, José-Roberto Viana-Silva, Paulo-Goberlânio de Barros-Silva, Eduardo-Costa Studart-Soares, Filipe-Nobre Chaves, Karuza-Maria Alves-Pereira, Thyciana-Rodrigues Ribeiro, Fábio-Wildson Gurgel-Costa

**Affiliations:** 1 DDS, MSc, PhD, Division of Oral Surgery, School of Dentistry, Fortaleza University (UNIFOR), Fortaleza, Brazil; 2 DDS, MSc, PhD, Division of Clinical Dentistry, Postgraduate Program in Dentistry, Federal University of Ceará, Fortaleza, Brazil; 3 MSc, PhD, Biotechnology Nucleus of Sobral – NUBIS, School of Medicine, Federal University of Ceará, Sobral, Brazil

## Abstract

**Background:**

This study aimed to evaluate the gene expression of cyclooxygenases (COXs) in an oral model of preemptive analgesia.

**Material and Methods:**

Gingival tissue was collected during extraction of lower third molars from a randomized, triple-blind, split-mouth and placebo-controlled study. The eligible patients were randomly sorted to receive a single dose either of ibuprofen 400mg, or etoricoxib 120 mg or a placebo, one hour prior to surgery. The temporal course of RNAm was evaluated for COX-1 and -2 by means of a quantitative polymerase chain reaction in real time (RT-qPCR) at time zero and 30 minutes after the surgical procedure began, and it was correlated with clinical parameters (pain and maximum mouth opening).

**Results:**

There was a significant increase in COX-1 expression between T0 and T30 in ibuprofen (*p*=0.004) and etoricoxib (*p*=0.010) groups. As regards COX-2, there were increases from T0 to T30 in all groups (placebo, *p*=0.012; ibuprofen, *p*<0.001; etoricoxib, *p*<0.001). All groups showed a significant decrease in COX-2:COX-1 ratio from T0 to T30 (placebo, *p*=0.013; ibuprofen, *p*<0.001; etoricoxib, *p*=0.047). Experimental groups showed a significant correlation between COX-1 and COX-2 levels and clinical pain parameters.

**Conclusions:**

The present preemptive analgesia study concludes that COX-2 RNAm induction was directly linked to third molar-related tissue inflammation and that the relation between COX-1 and COX-2 levels were inversely proportional to the preemptively administered nonsteroidal anti-inflammatory drugs COX-2 selectivity.

** Key words:**Preemptive analgesia, dental extraction, cyclooxygenases, real-time polymerase chain reaction.

## Introduction

Cyclooxygenase (COX) catalyzes the initial steps in the synthesis of prostaglandins (PGs) and other eicosanoids from arachidonic acid. PGE2, one of the many arachidonic acid metabolites derived from COX, is released in inflamed tissues, sensitizing afferent nerve fibers, and increasing nociception to evoke a hyperalgesic state (1). COX-1 and -2 are derived from different genes and constitute the main targets of non-steroidal anti-inflammatory drugs (NSAIDs). The messenger RNA (mRNA) originated from COX-1 expression presents a half-life of approximately 12-15 hours, whereas COX-2 gives rise to mRNA with a shorter half-life of less than 3.5 hours (2). These findings suggest an intrinsic temporal connection between tissue injury, COX-2 expression and the observed increase in PGE2 levels during inflammation. This connection is not observed in association with the constitutively expressed COX-1. COX inhibition provided by NSAIDs confers relief of pain and inflammation that follows oral surgery procedures, justifying clinical interest on COX isoforms (3).

Third molar surgeries are highly invasive procedures capable of triggering various levels of inflammatory pain that may potentially impact the quality of life of patients with short and medium-term repercussions; hence, these procedures have been historically established models to study the efficacy of various centrally and non-centrally acting analgesics and anti-inflammatory drugs (4-6). A previous study demonstrated a distinct synthesis of COX-1 metabolites and PGE2 production mediated by COX-1 and -2 following oral surgery procedures, in the absence of rescue medications (7).

Preemptive analgesia aims to prevent or diminish postoperative pain and inflammation, reducing the need for medication in the days immediately following surgery (3,8). Studies have demonstrated etoricoxib’s efficacy as a selective COX-2 inhibitor with few gastro-intestinal effects when used to treat acute pain associated with oral-dental surgery (3), and 120 mg was described as the minimum dose of etoricoxib that demonstrates maximum analgesic effect (9). In addition, ibuprofen is one of the most commonly used drug to control dental pain, and its efficacy in treating pain associated with dental surgery in the postoperative period has been widely demonstrated (8,10).

The primary objective in this study was to evaluate the association between COX-1 and COX-2 RNAm induction and clinical inflammatory parameters (pain scores, rescue medication intake, and maximum mouth opening) in third molar surgeries. This objective was based on the study hypothesis that the inflammatory process related to these surgical procedures is associated with COX-1 and COX-2 RNAm induction. In addition, the secondary objective in this investigation was to assess the levels of COX-1 and COX-2 RNAm according to the preemptively administered NSAID in third molars surgeries. This objective was formulated to test the hypothesis that the NSAID type used before the surgical procedure may influence the COX gene isoforms expression in gingival tissue collected from patients exposed to the preemptive administration of placebo, ibuprofen, and etoricoxib.

## Material and Methods

-Study Design and Sample size calculation

This study had an analytical design. Gingival tissue was collected during extraction of impacted lower third molars from patients, during the course of a previous clinical trial that had a randomized, triple-blind, split-mouth and placebo-controlled study design (11). The following inclusion criteria were adopted to standardize the level of traumatic injury generated by surgery: patients with third molars requiring ostectomy, with or without associated tooth sectioning; patients with third molars that showed similar patterns of root formation, position, and degree of impaction. In addition, the following exclusion criteria were adopted: smokers, pregnant or breast feeding, users of medications that could interact with the drugs used in this study, patients with orthodontic bands on the mandibular second molars, confirmed history of allergy to NSAIDs, signs of any preoperative inflammatory or infectious condition, systemic chronic disease, use of NSAIDs within the past 21 days, or the presence of periodontal disease, swelling, fever, or trismus prior to surgery (11). The previous study was approved by the Ethics Committee of the Walter Cantídio University Hospital (WCUH) No. 44058715.4.0000.5045.

During that study, patients donated tissue for the present investigation by signing an informed consent form. Patients had been subjected to preemptive analgesia by taking ibuprofen 400mg, or etoricoxib 120mg or a placebo with no active pharmaceutical principal. In addition, the previously recorded pain scores by using the visual analog scale (VAS) at 0, 2, 4, 6, 8, 10 and 12 hours, and 1, 5 and 7 days postoperatively, rescue medication intake, maximum mouth opening (at baseline and 7 days postoperatively), and the surgical period duration were evaluated in the present research.

A previous study (3) observed VAS of 2.7±1.6 and 0.2±0.1 for two different groups preemptively treated with placebo and etoricoxib, respectively. The data obtained established that a minimum sample size of 5 surgical sites per group yields a power of 90%, and alpha=0.05 in order to accept or reject the null hypotheses. To obtain the minimum sample size per group from the original study carried out by Albuquerque *et al.* (11) a second randomization was performed using a method to generate the random allocation sequence (“randomization per block” function of the Microsoft Excel®).

-Sample acquisition and Study of the time-course of COX-1 and COX-2 mRNA expressions

This study sample consisted of 30 fragments of pericoronal tissue evenly distributed according to treatment received (ibuprofen, n=10; etoricoxib, n=10; placebo, n=10), and time of collection per group (T0, n=5 per group and T30, n=5 per group). Gingival fragments of pericoronal tissue (close to the tooth being removed) were collected in two separate moments (T0= at the beginning of surgery and T30= 30 minutes later). Samples were identified by a number so that the investigator would not know which group gingival samples belonged to.

To study the time-course of the COX-1 and COX-2 gene expression, the primers were designed on the basis of data obtained from the NCBI gene bank using the PrimerBlast program with exclusive specificity for Homo sapiens. GAPDH was used as the endogenous control (housekeeping) gene because it is a gene that is not affected by the inflammatory condition that is being analyzed in the present study and also to normalize samples for possible differences in cDNA quantities added in each reaction. The primers used for the target genes (COX-1 and COX-2) were developed by exon-exon ligating, thereby making genomic DNA amplification unfeasible.

-Spectrophotometric Quantification 

To test the efficacy of extraction and total RNA purity the concentration of total RNA in the samples was determined by RNA dilution (known dilution factor) together with a spectrophotometric reading in quartz cuvettes, using wavelengths of de 260 nm (A260) and 260/280 nm (A260/A280).

-RNA extraction and cDNA synthesis

Isolation of total RNA was performed using the PureLink® RNA Mini Kit (Life Technologies, New York, USA). The RNA concentration was estimated by reading the absorbance at 260 nm and was checked for purity at 280 nm in a spectrophotometer (Amersham Biosciences, Cambridge, England). For each sample, RNA concentrations were adjusted and used to synthesize cDNA with 1 µL. Before the reverse transcription reaction, samples of RNA were incubated for 5 min at 70 ºC and then cooled in ice. The reverse transcription was performed in a total volume of 20 µL composed of 10 µL of sample RNA, 4 µL reverse transcriptase buffer (Invitrogen, São Paulo, Brazil), 8 units RNase out, 150 units of reverse transcriptase Superscript III, 0036 U random primers, 10 mM DTT and 0.5mM of each dNTP (Invitrogen, São Paulo, Brazil). The mixture was incubated at 42 ºC for 1 h, subsequently at 80 ºC for 5 min, and finally stored at –20 ºC. The negative control was prepared under the same conditions, but without the addition of reverse transcriptase.

-Quantitative reverse transcription (qRT)-PCR evaluation

Quantification of mRNA was performed using SYBR GreenMaster Mix (PE Applied Biosystems, Foster City, CA). PCR reactions were composed of 1 μL cDNA as a template in 7.5 μL of GoTaq® qPCR Master Mix (Promega Corporation, Madison, WI, USA), 5.5 µL of ultra-pure water, and 0.5 μM of each primer. The primers were designed by using the PrimerQuestSM program (http://www.idtdna.com), and GAPDH was used as the normalizing gene. The specificity of each primer pair was confirmed by melting curve analysis of PCR products. The thermal cycling profile for the first round of PCR was: initial denaturation and activation of the polymerase for 10 min at 95 oC, followed by 40 cycles of 15 sec at 95 ºC, 30 sec at 58 ºC, and 30 sec at 72 ºC. The final extension was for 10 min at 72 ºC. All reactions were performed in StepOne Real-Time PCR, and relative quantifications of mRNA used the comparative threshold cycle (Ct) (Ct) method.

-Statistical Analysis

Data were expressed in mean and standard error of the mean for comparison with pared t-test or ANOVA (1-way or 2-way) followed by Bonferroni post hoc test. Chi-square and Fisher Exact tests were used to evaluate associations between categorical variables (n, %) (GraphPad Prism 5.0, *p*<0.05). Pearson correlation was used in order to correlate COX-1 and COX-2 levels with clinical parameters.

## Results

-Sample characterization and mRNA expression of COX-1 and COX-2

**Figure 1:**
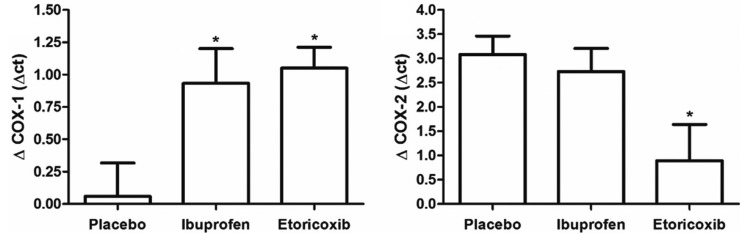
COX-1 and COX-2 tissue level between the studied groups at 0 and 30 minutes after the surgical procedure. *p<0.05 in relation to the experimental period 0 minutes of the same group (paired t-test).

The average age of the patients was 22 years. Patients did not differ regarding demographic or surgical factors, such as the eventual extraction difficulties, dental position, or quantity of anesthetic used (Table 1). RT-PCR showed no difference in COX-1 expression in the placebo group from T0 to T30; however, in the groups treated with ibuprofen (*p*=0.004) and etoricoxib (*p*=0.010) showed a significant increase in the COX-1 expression from the first (T0) to the second moment (T30) (Fig. 1A). The increase in the COX-1 expression was significantly greater in the groups treated with ibuprofen (0.9±0.3) and etoricoxib (1.1±0.2) than in the placebo group (0.1±0.2) (*p*=0.020).

**Table 1:**
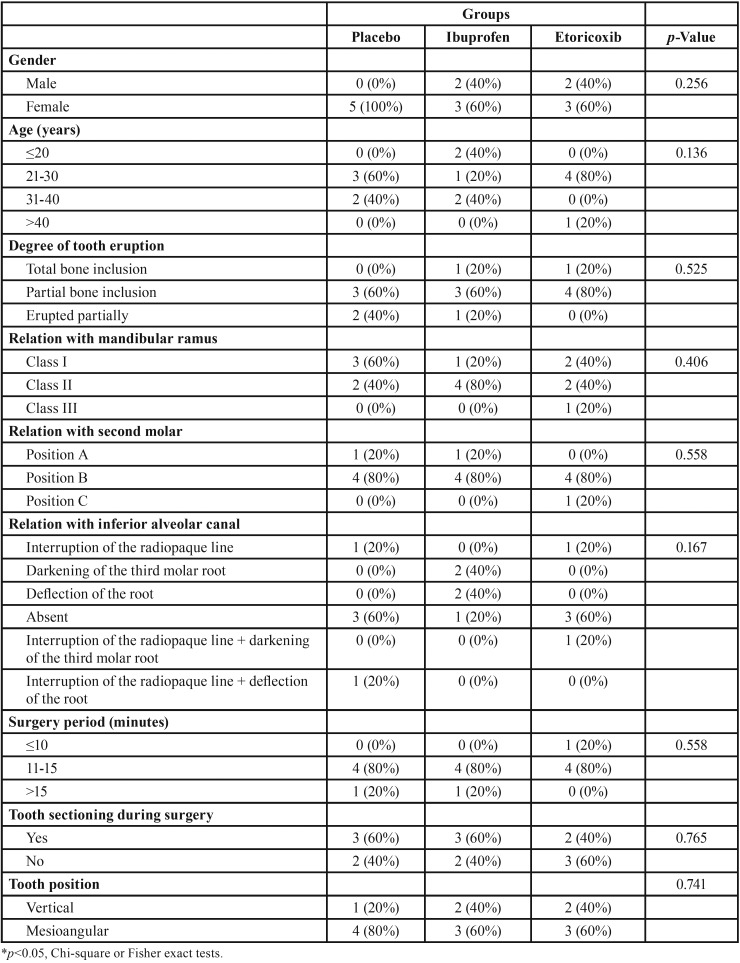
Sample characterization.

**Figure 2:**
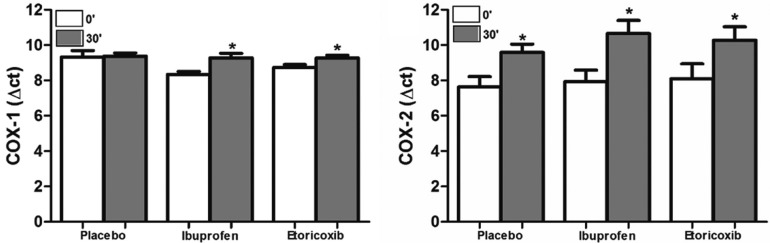
Variation of the COX-1 and COX-2 tissue level between the studied groups after the surgical procedure. *p<0.05 in relation to the experimental groups versus the placebo group (one-way ANOVA test).

All three groups showed an increase in COX-2 expression from T0 to T30 (placebo, *p*=0.012; ibuprofen, *p*<0.001; etoricoxib, *p*<0.001) (Fig. 1B). Only the group treated with etoricoxib showed a modest increase in COX-2 expression compared to the placebo group (*p*=0.023); however, there was no difference between the placebo group and the group treated with ibuprofen (Fig. 2). The three groups showed a significant reduction in the ratio of COX-2 to COX-1 expressions from T0 to T30 (placebo, *p*=0.013; ibuprofen, *p*<0.001; etoricoxib, v=0.047).

-Relationship between COX-1 and COX-2 expressions and clinical parameters

**Figure 3:**
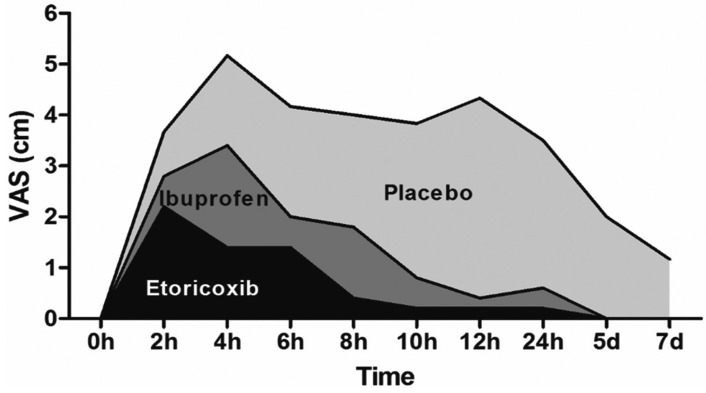
Graphical representation of the pain scores over the studied periods. The area under the curve shows pain experience significantly reduced in etoricoxib (*p*<0.0001) and ibuprofen (*p*=0.006) groups in comparison with placebo, and significantly reduced in etoricoxib group in comparison with ibuprofen group (*p* = 0.0488). P, placebo; two-way ANOVA test.

Clinically, pain scores of the ibuprofen group were significantly lower than the placebo group from 8h to 24h after the surgical procedure (*p*<0.001). The pain scores of the etoricoxib group were significantly lower in comparison with the placebo group from 4h to 24h after the surgical procedure (*p*<0.001), and pain scores of the etoricoxib group were significantly lower in comparison with the ibuprofen group 4h after the surgical procedure (*p*=0.047). The area under the postoperative pain experience curve of the placebo group (31.2) was 2.6 times higher than the ibuprofen group (11.8) and 5.2 times higher than the etoricoxib group (6) (Fig. 3). In relation to the maximum mouth opening 7 days after surgery, there was a statistical difference (*p*=0.001) between placebo (11.5±1.9 mm), ibuprofen (4.4±0.7 mm), and etoricoxib (2.4±0.6 mm) groups.

According to  Table 2- Table 4, group treated with ibuprofen showed an inverse correlation between COX-1 level at T0 and pain peak after 4h (*p*=0.034, r= -0.905), between COX-1 level at T30 and baseline mouth opening (*p*= 0.044, r = -0.889) and after 7 days (*p* = 0.013, r = -0.915). There was also a significant inverse correlation between COX-2 level at T0 and pain peak after 10h (*p*=0.001, r = -0.990) and 12h (*p*=0.001, r = -0.990), and T30 and pain peak after 24h (*p*=0.001, r = -0.993). In addition, COX-2 level and the consumption of rescue medication were directly correlated (*p*=0.001, r = 0.990). In the group treated with etoricoxib, there was a significant inverse correlation between COX-1 level at T0 and pain peak after 2h (*p*=0.015, r = -0.947), as well as direct correlation between COX-1 and pain peak after 6h (*p*=0.032, r = 0.910). COX-2 level showed a significant direct correlation with pain peak after 24h in both T0 (*p*=0.006, r = 0.969) and T30 (*p*=0.027, r = 0.919) evaluated periods.

**Table 2:**
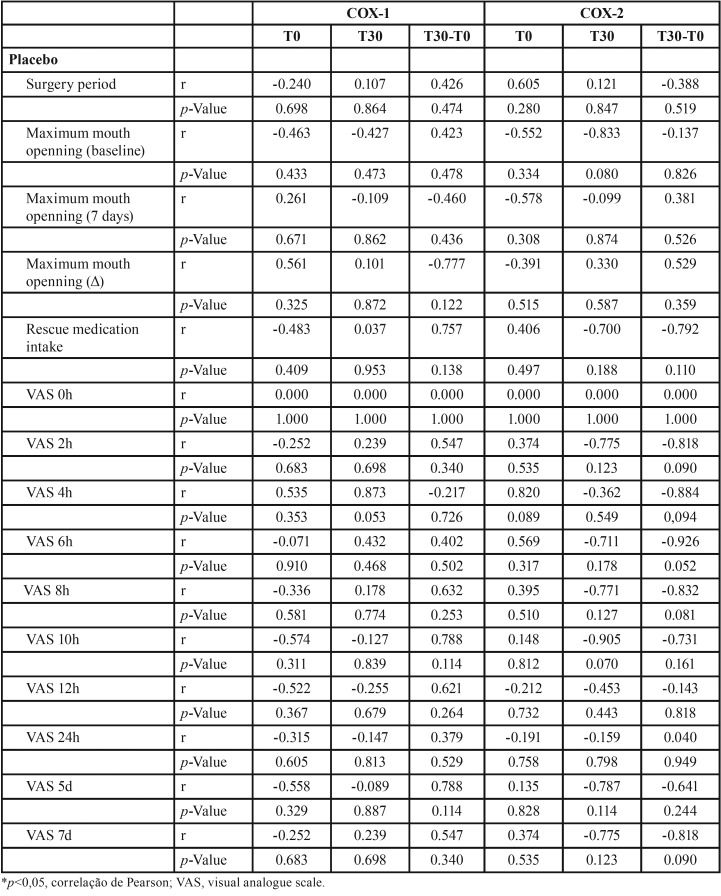
Pearson correlation between COX-1 and COX-2 gene expressions and clinical parameters (placebo group).

**Table 3:**
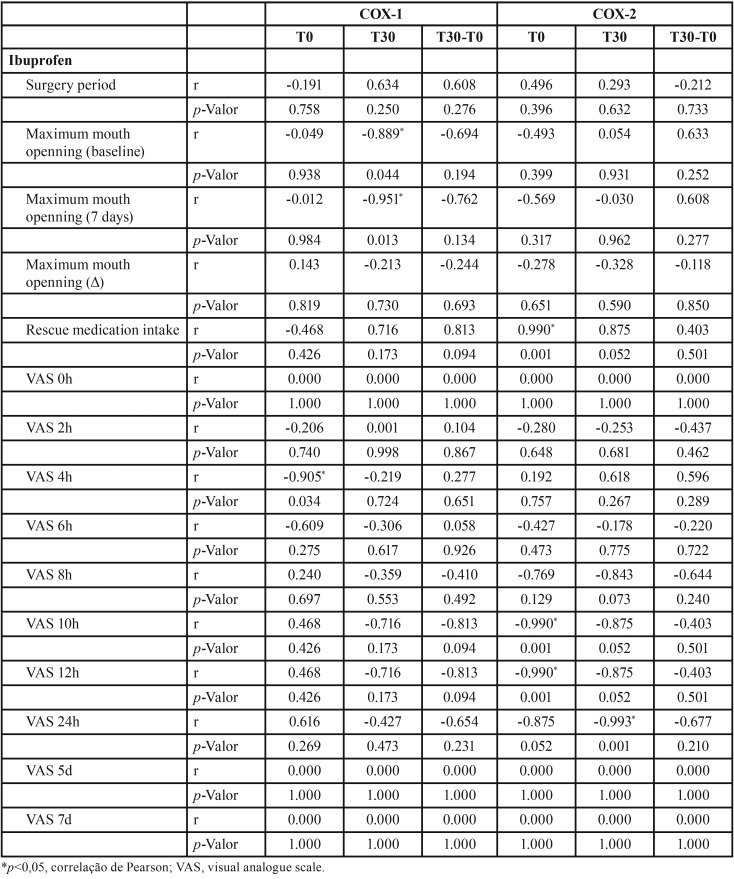
Pearson correlation between COX-1 and COX-2 gene expression and clinical parameters (ibuprofen group).

**Table 4:**
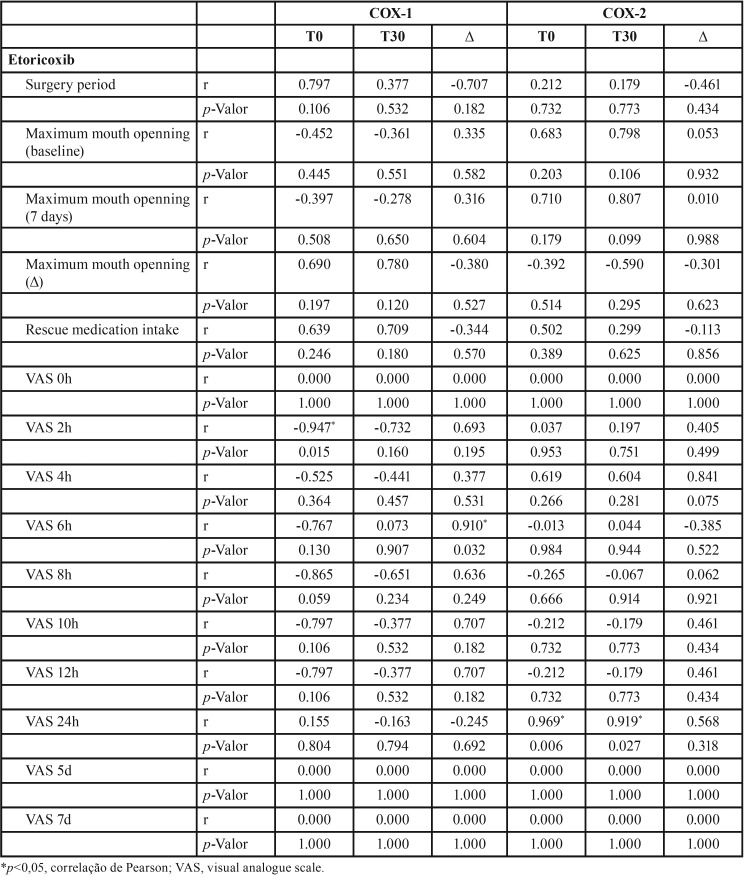
Pearson correlation between COX-1 and COX-2 gene expression and clinical parameters (etoricoxib group).

## Discussion

Third molar surgery was selected to validate the clinical model used in the research because it has been widely practiced and validated in pharmacological trials since 1976 by Cooper and Beaver (12), as well as being a common dental procedure in which postoperative pain is usually short-lasting reaching its height in the initial stage immediately after the surgical trauma affecting the surrounding tissues (13). That model has been considered highly important in clinical investigations to distinguish the analgesic effects of various drugs, as was the case in the present research, or to investigate the effects of different dosages of a single drug (14,15).

This study investigated the effect of preemptive oral administration of ibuprofen and etoricoxib on COX-1 and COX-2 levels in gingival tissue. These two drugs are commonly administered in lower third molar removal procedures as a means of controlling postoperative pain. In fact, the area under the curve, correlating the pain scores over the time, showed that both experimental groups reduced the pain scores in comparison with the placebo group, and the etoricoxib was the drug who significantly reduced the pain scores. Cyclooxygenase, also known as prostaglandin H synthase is the key enzyme in prostaglandin synthesis. The original elucidation of the two COX isoforms gave rise to the concept that the constitutive enzyme COX-1 was responsible for the production of prostaglandins with homeostatic functions in stomach and kidney tissues and in platelet aggregation, whereas COX-2 is induced and responsible for the production of pro-inflammatory substances, especially PGE2 (16). The contribution of COX-2 to inflammation is further supported by the fact that COX-2 expression can become from ten to 80 times greater in the presence of pro-inflammatory cytokines such as IL-6, IL-1, and prostaglandin production can be inhibited by anti-inflammatory cytokines (17). However, most studies have focused on measuring tissue cytokine levels instead of evaluating the impact of the use of COX-2 selective NSAIDs on tissue levels of these enzymes. To our knowledge this is the first investigation evaluating gene expression of COXs in human tissues following third molar surgery from a split-mouth study, concomitantly evaluating the preemptive analgesic effect of etoricoxib and ibuprofen.

Khan *et al.* (7) conducted a similar clinical study that used the same surgical procedures and collected gingival specimen in patients underwent third molar surgery without preoperative administration of NSAIDs aiming to evaluate COX expression in oral tissues without the use of medication. The aforementioned study showed a gradual increase in COX-2 expression at 30, 60, and 120 minutes after surgery, which is expected for those patients that did not intake any NSAIDs. For COX-1, however, there was a slight drop at 30 minutes and a significant reduction at 60 minutes, but by 120 minutes, the COX-1 expression returned to initial levels. However, it is difficult to make any comparisons between their study and the present one because each temporal analysis was carried out with a different patient. In other words, no single patient was subsequently analyzed at three times so that there is no way of knowing whether the data would have maintained the same pattern had it been registered for a single patient on all occasions. In comparison with the present study design, there were no medications investigated in a previous gene expression study (7). If there is a potential change in COX expression-related parameters following an inflammatory process such as dentoalveolar surgeries, these data could be properly evaluated in third molar studies involving NSAIDs as presently performed. In the present investigation, COX-1 levels in the placebo group did not differ between T0 and T30, differing from Khan *et al.* (7) that observed a slight COX-1 level decrease in the studied groups. In the ibuprofen and etoricoxib groups, however, there was a slight increase that is believed by the use of those drugs as the reduction in COX-2 could lead to compensatory expression of COX-1.

No significant differences were detected in the COX-2/COX-1 ratio among the three studied groups. That can be explained by cascade compensations and the selectivity of the medications. A high level of COX-1 and COX-2 was observed in the placebo group, whereas in the etoricoxib and ibuprofen groups due to the reduction in COX-2 expression and the increase in COX-1 expression, there were no observable differences in the COX2/COX1 ratios. That compensatory behavior shows that even non-selective drugs can have satisfactory analgesic and anti-inflammatory effects (17), which is supported by our findings since the Pearson correlation showed a statistically significant difference in the experimental groups. Ibuprofen group showed an opposite correlation between specific pain peaks and gene expression of both COXs, an opposite relationship between maximum mouth opening and COX-1 expression, and a direct correlation between COX-2 expression and rescue medication intake. Etoricoxib group showed an opposite correlation between the COX-1 gingival level and specific pain peaks and a direct correlation between COX-2 level and determined pain scores.

In third molar studies evaluating gene expression of COXs along with the preemptive use of NSAIDs (19,20), it is possible to observe results that corroborate with the present findings regarding the temporal expression of COXs. Lee *et al.* (19) showed a COX-1 expression decrease (36%) after 2 and 4h postoperatively, and a significant COX-2 expression increase (300%). The test groups (ibuprofen and rofecoxib) had a significant increase in COX-2 when compared to the placebo group. In addition, these authors showed a significant relationship between gene polymorphism variability and patient pain relief after the use of NSAIDs. In the study performed by Lee *et al.* (20), ketorolac decreased COX-1 gene expression at the 24-h postoperative evaluation and suppressed TBX2. Also, PGE2-related COX-2 expression remained high even in the 3-h and 24-h periods. These findings showed that the effect of COX-selective inhibitors on PGE2 levels contribute to inflammatory pain relief after third molar surgery, which may support the present results.

## Conclusions

The present preemptive analgesia study concludes that that COX-2 RNAm induction was directly linked to third molar-related tissue inflammation and that the relation between COX-1 and COX-2 levels were inversely proportional to the preemptively administered NSAID COX-2selectivity. Clinically, COX-1 and COX-2 gene expressions were correlated with third molar-related inflammatory events, notably the pain parameters.
